# Cutaneous Basal Cell Carcinoma In Situ: A Case Series

**DOI:** 10.7759/cureus.29479

**Published:** 2022-09-23

**Authors:** Philip R Cohen

**Affiliations:** 1 Dermatology, University of California, Davis Medical Center, Sacramento, USA

**Keywords:** squamous cell carcinoma in situ, squamous cell carcinoma, skin, melanoma in situ, melanoma, in situ, cutaneous, cancer, basal cell carcinoma in situ, basal cell carcinoma

## Abstract

Basal cell carcinoma, squamous cell carcinoma, and melanoma are three types of skin cancers. Skin cancers present as either non-invasive malignancies or invasive neoplasms. The non-invasive malignancies are referred to as carcinoma in situ; they only have cancer cells that are restricted to the epidermis and are not present as single tumor cells or independent nests of malignant cells in the underlying dermis. In contrast, invasive neoplasms demonstrate individual malignant cells or aggregates of tumor cells or both that are found in the dermis; in addition, cancer cells may also be located in the overlying epidermis. The features of three men with basal cell carcinoma in situ of the skin are described in this case series. Each of their tumors morphologically appeared as a red, scaly or non-scaly, and superficial plaque on the abdomen or the back; the neoplasm was successfully treated without recurrence by either surgical excision or by topical 5% imiquimod cream. Cutaneous basal cell carcinoma in situ has characteristic morphologic and pathologic findings. Clinically, cancer most commonly appears as a thin, smooth-surfaced or scaly, and as an erythematous plaque on the trunk. Microscopic examination shows multiple sites of basaloid tumor cells that either nearly filled the epidermis and/or replaced the lower layers of the epidermis; the aggregates of tumor cells may expand the epidermis and display slight extension into the papillary dermis. However, there is no non-contiguous invasion of the tumor cells from the overlying epidermis into the underlying dermis. Palisading of the peripheral tumor cells is noted; in addition, focally, retraction of the dermal stroma from the tumor is also frequently present. Lymphocytic inflammation may also be present in the upper dermis. Similar to the reported patients, since basal cell carcinoma in situ of the skin is only localized to the epidermis, several therapeutic modalities are available to effectively treat the cancer. However, if the basal cell carcinoma in situ represents the superficial portion of a basal cell carcinoma of mixed histology, tumor persistence or recurrence may occur if a more conservative approach to treatment is utilized that does not adequately treat the unsuspected and more aggressive pathologic subtype of basal cell carcinoma in the underlying dermis. In summary, basal cell carcinoma in situ of the skin has a unique clinicopathologic correlation of morphology, histology, tumor biology, and response to treatment; indeed, tumors that were previously classified as superficial basal cell carcinoma are more appropriately designated as cutaneous basal cell carcinoma in situ.

## Introduction

Cancer includes hematologic malignancies and solid tumors. Solid tumors are often referred to as carcinomas. Carcinomas include skin cancers such as basal cell carcinoma and squamous cell carcinoma [1-5].

Invasion of malignant cells is a characteristic of cancer. Tumor invasion, a pathognomonic feature of a cutaneous carcinoma, may be defined as the presence of the neoplasm - that is not contiguous with the overlying epidermis - in the dermis. In contrast, in situ carcinoma refers to skin cancer in which the malignant cells are restricted to the overlying epidermis without non-contiguous invasion into the underlying dermis [1-7].

Basal cell carcinoma is the most common skin cancer. There are several morphologic presentations of basal cell carcinoma; each has pathologic findings that correlate with the tumor’s clinical appearance. In this case series, three men with cutaneous basal cell carcinoma in situ are described and the features of basal cell carcinoma in situ of the skin are discussed.

## Case presentation

Case 1

A 43-year-old Fitzpatrick skin type 1 Caucasian male presented with an asymptomatic lesion on his right torso of six months duration. It had begun as a small red spot and had continued to slowly enlarge. He also had a history of biopsy-confirmed seborrheic keratosis; however, he had no previous skin cancers.

**Figure 1:**
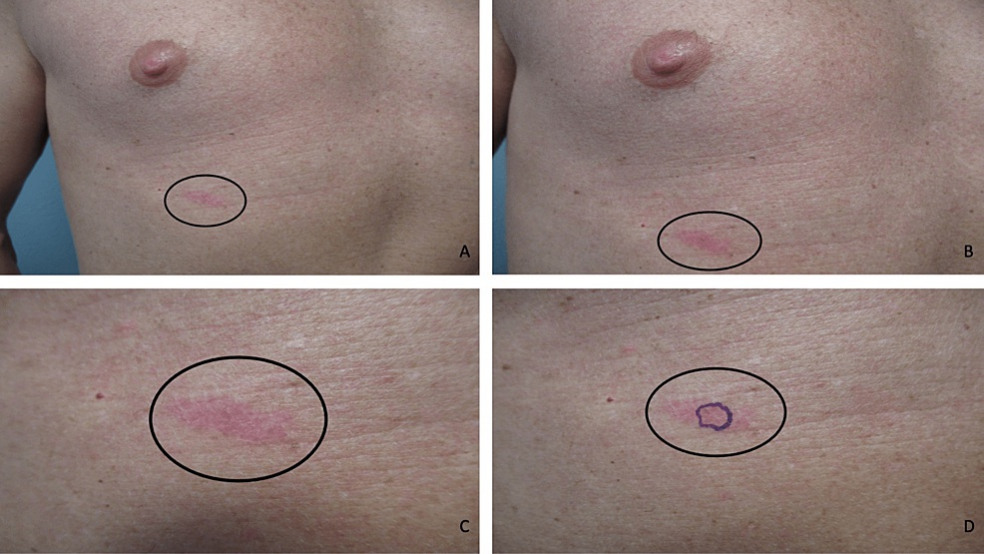
Morphologic features of a cutaneous basal cell carcinoma in situ. Distant (A) and closer (B, C, and D) views of a cutaneous basal cell carcinoma in situ presenting as an asymptomatic, red, triangular-shaped 5 x 4 x 4 cm plaque (black oval) on the right upper abdomen of a 43-year-old Fitzpatrick skin type 1 Caucasian male. The biopsy site is designated by the purple oval (D).

A complete cutaneous examination was done. A painless and non-pruritic, erythematous, triangular-shaped 5 x 4 x 4 cm plaque was observed on his right upper abdomen (Figures 1A-1D). A partial biopsy of the skin lesion using the shave technique was performed.

**Figure 2:**
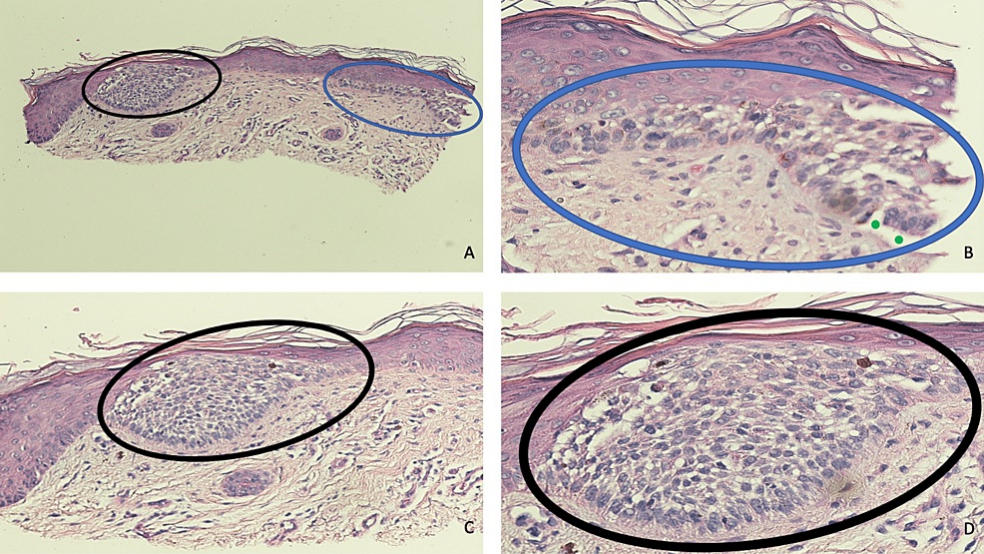
Pathologic features of a basal cell carcinoma in situ of the skin. Distant (A) and closer (B, C, and D) views of microscopic findings of a cutaneous basal cell carcinoma in situ. Large aggregates of basaloid tumor cells (black ovals) nearly filled the entire epidermis and extended downward into the papillary dermis (A, C, and D). Malignant tumor cells (blue ovals) also replaced the lower layers of the epidermis with slight extension into the underlying dermis (A and B). There is palisading of the peripheral tumor cells (B and D). Importantly, non-contiguous invasion of the tumor cells into the underlying dermis is absent. Retraction of the dermal stroma from the tumor (solid green circles) is also focally present (B) (hematoxylin and eosin: A, x10; B, x40; C, x20; D, x40).

Microscopic evaluation of the tissue specimen showed several collections of basaloid tumor cells that either nearly filled the epidermis and/or replaced the lower layers of the epidermis; the aggregates of tumor cells expanded the epidermis with slight extension into the papillary dermis. However, there was not any non-contiguous invasion of the tumor cells into the underlying dermis. Palisading of the peripheral tumor cells was noted; in addition, focally, retraction of the dermal stroma from the tumor was present (Figures 2A-2D).

Correlation between the clinical presentation and the pathology findings established the diagnosis of basal cell carcinoma in situ. The residual tumor was excised. There has been no recurrence of the neoplasm after 15 months of follow-up.

Case 2

A 63-year-old Fitzpatrick skin type 1 Caucasian male presented with a red lesion on his right flank of four months duration. It was a slow-growing lesion that began as a small bump on his skin. He has had several squamous cell carcinomas that have been excised and prior basal cell carcinomas that have either been excised or successfully treated topically with imiquimod cream.

**Figure 3:**
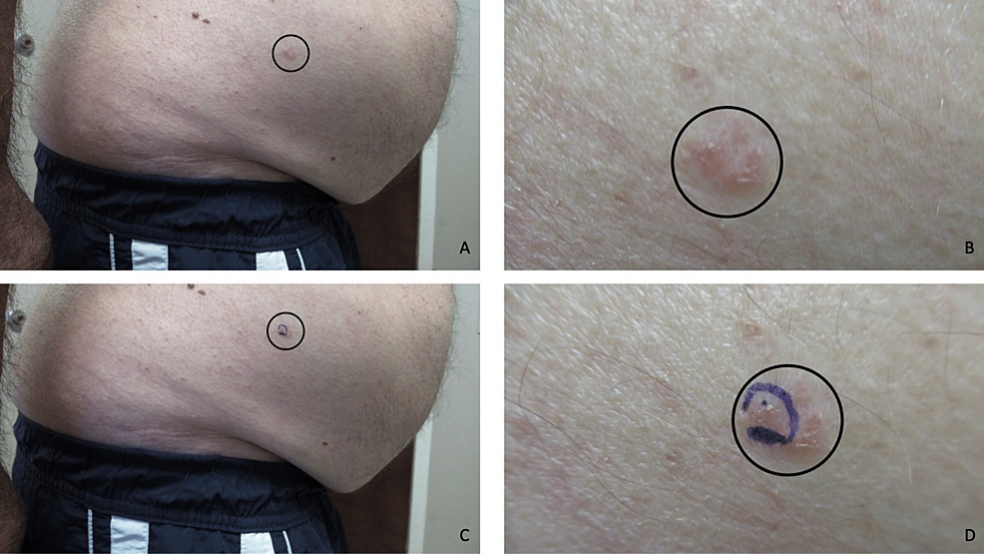
Clinical presentation of a basal cell carcinoma in situ of the skin. Distant (A and C) and closer (B and D) views of a basal cell carcinoma in situ of the skin that presented as a slowly growing, painless and non-pruritic, red, scaly 2 x 2 cm plaque with peripheral nodules (black oval) on the lateral right lower abdomen of a 63-year-old Fitzpatrick skin type 1 Caucasian male. The patient has had several prior skin cancers that were previously treated; his squamous cell carcinomas were completely excised and his basal cell carcinomas were successfully treated either by surgical excision or topical application of 5% imiquimod cream. Using the shave technique, a partial biopsy of his new skin lesion (purple oval) was performed (C and D).

A complete cutaneous examination was done. A non-tender and itch-free, erythematous, 2 x 2 cm scaly plaque with peripheral nodules was noted on his lateral right lower abdomen (Figures 3A-3D). A partial biopsy of the skin lesion using the shave technique was performed.

**Figure 4:**
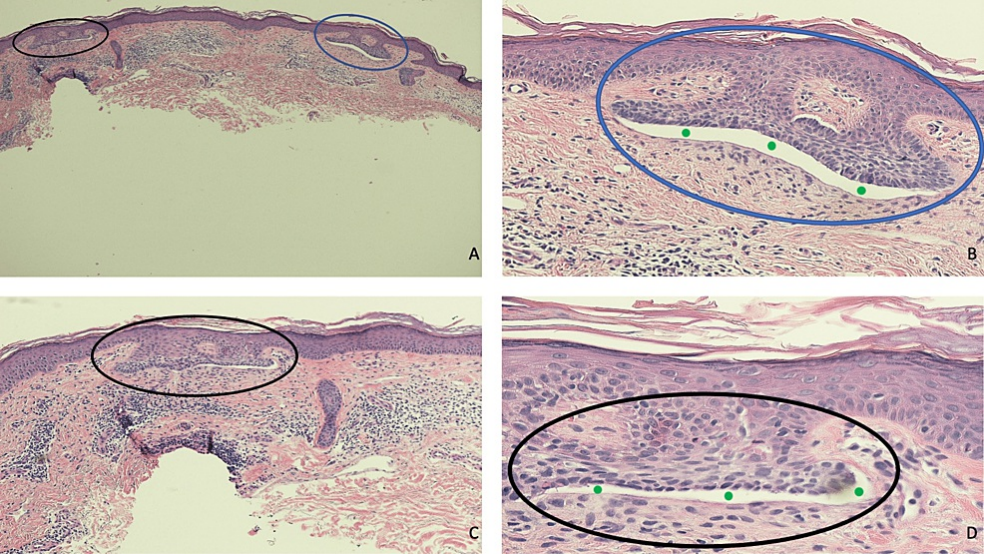
Microscopic presentation of a cutaneous basal cell carcinoma in situ. Distant (A) and closer (B, C, and D) views of microscopic findings of a cutaneous basal cell carcinoma in situ of the skin. Contiguous extension of the epidermis, which contains basaloid tumor cells, into the underlying papillary dermis (black ovals and blue ovals) can be observed at several sites (A, B, C, and D); however, non-contiguous invasion of the tumor cells into the underlying dermis is not present. The malignant tumor cells (black and blue ovals) are predominantly restricted to the lower layer of the epidermis (A, B, C, and D); also, at the periphery of the tumor aggregates (black and blue ovals), there is palisading of the neoplastic cells (B and D). A clear cleft (solid green circles) is created by the retraction of the dermal stroma adjacent to the tumor from the peripheral cells of the neoplasm (B and D). In the upper dermis, several areas contain lymphocytic inflammation (A, B, C, and D) (hematoxylin and eosin: A, x4; B, x20; C, x10; D, x40).

Microscopic evaluation of the tissue specimen showed several areas that have contiguous extension of the epidermis into the underlying papillary dermis. Predominantly restricted to the lower layer of the epidermis are basaloid tumor cells with palisading of the cells at the periphery; there is not any non-contiguous invasion of the tumor cells into the underlying dermis. The dermal stroma adjacent to the tumor is retracted from the peripheral cells of the neoplasm. There are several focal areas of dense inflammation composed of lymphocytes in the upper dermis (Figures 4A-4D).

Correlation between the clinical presentation and the pathology findings established the diagnosis of basal cell carcinoma in situ. The residual tumor was treated with topical 5% imiquimod cream applied to the remaining cancer and surrounding skin for five consecutive nights each week for six weeks. The treatment site became inflamed; after the six weeks of treatment was completed, the inflammation was resolved and there was no residual tumor. There has been no recurrence of the skin malignancy after 21 months of follow-up.

Case 3

A 69-year-old Fitzpatrick skin type 1 Caucasian male presented for a skin check. He was not aware of any new lesions. He had no previous skin cancers.

**Figure 5:**
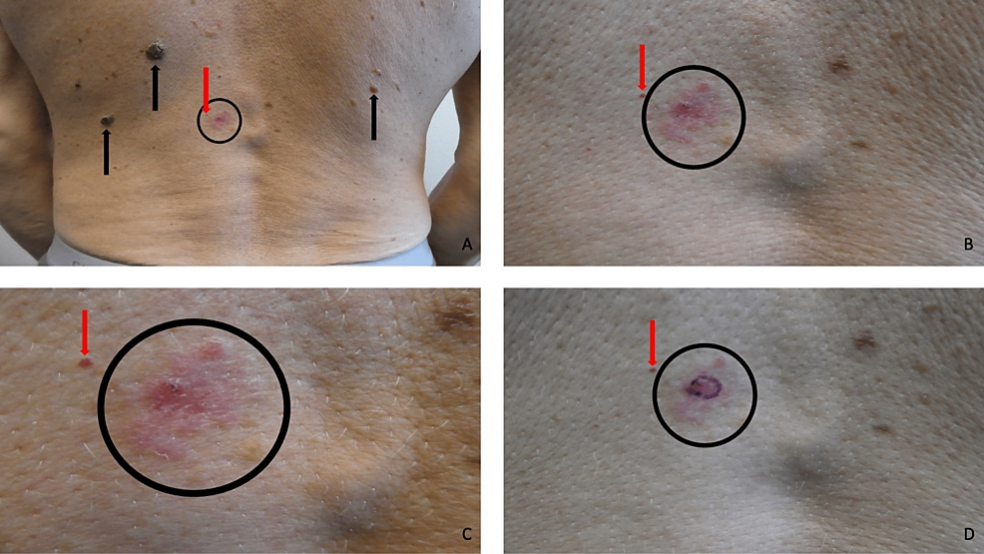
Cutaneous basal cell carcinoma in situ presenting as an asymptomatic plaque on the back. Distant (A) and closer (B, C, and D) views of a cutaneous basal cell carcinoma in situ appearing as a non-tender, red, focally crusted 4 x 2.5 cm plaque with peripheral nodules (black ovals) were observed on the left lower back of a 69-year-old Fitzpatrick skin type 1 Caucasian male. The purple oval shows the site of a partial biopsy of the skin lesion using the shave technique (D). Additional lesions are also on his back - seborrheic keratoses (black arrows) presenting as brown plaques (A) and a hemangioma (red arrow) presenting as a red papule (B, C, and D).

A complete cutaneous examination was done. An asymptomatic, erythematous, focally crusted 4 x 2.5 cm plaque with peripheral nodules were observed on his left lower back. In addition, three brown plaques (consistent with seborrheic keratoses) and a red papule (consistent with a hemangioma) were also present on his back (Figures 5A-5D). A partial biopsy of the skin lesion using the shave technique was performed.

**Figure 6:**
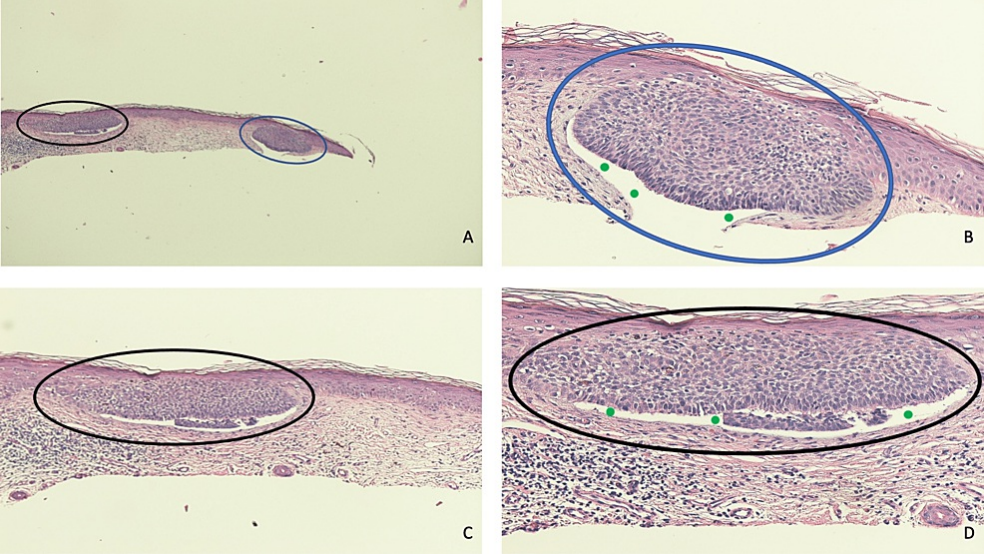
A basal cell carcinoma in situ of the skin with characteristic pathologic features. Distant (A) and closer (B, C, and D) views of pathognomonic microscopic findings of a cutaneous basal cell carcinoma in situ. The malignant cells (black ovals and blue ovals) partially replace the overlying epidermis and extend from the lower layers of the epidermis into the papillary dermis (A, B, C, and D). Also, the basaloid tumor cells (black ovals and blue ovals) are contiguous with the epidermis; importantly, non-contiguous invasion of either individual cancer cells or discrete nests of neoplastic cells into the underlying dermis is not present (A, B, C, and D). The peripheral tumor cells (black oval and blue oval) demonstrate palisading and the retraction of the dermal stroma from the margins of the tumor creates a clear cleft (solid green circles) between the periphery of the neoplasm and the surrounding dermis (B and D). In the upper dermis beneath the tumor, dense inflammation composed of lymphocytes is present (A, C, and D) (hematoxylin and eosin: A, x2; B, x4; C, x10; D, x20).

Microscopic evaluation of the tissue specimen showed several aggregates of basaloid tumor cells that are contiguous with the epidermis. The tumor cells extend from the lower layers of the epidermis into the papillary dermis and they partially replace the overlying epidermis. Retraction of the dermal stroma from the margins of the tumor is present and the peripheral tumor cells demonstrate palisading. There is no non-contiguous invasion of the tumor cells into the underlying dermis. Focally, there is lymphocytic inflammation in the upper dermis beneath the tumor (Figures 6A-6D).

Correlation between the clinical presentation and the pathology findings established the diagnosis of basal cell carcinoma in situ. The remaining tumor was excised. There has been no recurrence of cancer after two years of follow-up.

## Discussion

The most frequently occurring skin cancers include basal cell carcinoma, squamous cell carcinoma, and melanoma (Table 1) [1-7]. Cutaneous neoplasms may present as an in situ carcinoma or an in situ melanoma in which the tumor cells only occupy the superficial epithelium without individual tumor cells or aggregates of cancer cells independently being present in the deeper tissue. However, when the malignant cells that are not contiguous with the overlying epidermis or mucosa invade into the underlying dermis or submucosa, the cancer is referred to as carcinoma or melanoma [1-7].

**Table 1:** Types of skin cancers. ^a^The tumor cells are restricted to the epidermis and/or contiguous with the epidermis and extend into the papillary dermis. ^b^At least some of the tumor cells are non-contiguous with the overlying epidermis and are present as either individual cells or aggregates of cells in the dermis. ^c^This neoplasm was previously referred to as superficial basal cell carcinoma. ^d^Several basal cell carcinoma subtypes exist; some of these include basosquamous (metatypical), fibroepithelioma of Pinkus, giant, infiltrative, infundibulocystic, micronodular, morpheaform, nodular, pigmented, red dot, and ulcerative. ^e^When this neoplasm occurs on sun-exposed skin, it has been referred to as Bowen’s disease. ^f^Several squamous cell carcinoma subtypes exist; some of these include acantholytic, adenosquamous, clear cell, desmoplastic, keratoacanthoma, lymphoepithelioma-like, pseudovascular (pseudoangiomatous), spindle-cell (pseudosarcomatous or sarcomatoid), and verrucous (epithelioma caniculatum and oral florid papillomatosis). ^g^When this neoplasm occurs on sun-exposed skin, it has been referred to as lentigo maligna. ^h^Several melanoma subtypes exist; some of these include acral lentiginous, amelanotic, desmoplastic, lentigo maligna, nevoid, nodular, polypoid, superficial spreading, and verrucous.

Cell type	Non-invasive^a^	Invasive^b^	References
Basal cells	Basal cell carcinoma in situ^c^	Basal cell carcinoma^d^	[1-3]
Keratinocytes	Squamous cell carcinoma in situ^e^	Squamous cell carcinoma^f^	[4,5]
Melanocytes	Melanoma in situ^g^	Melanoma^h^	[6,7]

Pathologic features of squamous cell carcinoma in situ often show thickening of the epidermis with atypical neoplastic keratinocytes present in all layers of the epithelium. When the neoplasm is located in a sun-exposed area, it has been designated Bowen’s disease. Squamous cell carcinoma has several histologic variants; however, in all cancers, tumor keratinocytes that are not directly connected to the overlying epidermis are at least present in the papillary and/or deeper reticular dermis [4,5].

Similar to squamous cell carcinoma in situ, melanoma in situ shows a proliferation of atypical melanocytes restricted to the epithelium. They are either along the basal layer of the epidermis, or demonstrate an upward migration - referred to as a pagetoid spread - from the lower layers into the upper layers of the epidermis, or both. The presence of tumor melanocytes that are not contiguous with the overlying epidermis into the underlying dermis represents invasive melanoma. When melanoma in situ occurs on sun-exposed areas of the body, it is designated as lentigo maligna and the corresponding invasive cancer is called lentigo maligna melanoma [6,7].

Basal cell carcinoma has several distinctive clinical phenotypes. Each of these morphologic presentations of the tumor has specific pathologic features that characterize the neoplasm. Some of these subtypes include infiltrative basal cell carcinoma, morpheaform basal cell carcinoma, and nodular basal cell carcinoma [1-3,8].

Nodular basal cell carcinoma is the most prevalent form of basal cell carcinoma. It appears as a pearly and shiny, flesh-colored, smooth-surfaced, and telangiectatic papule or nodule that is often located on the head and neck. Microscopic examination shows discrete nodular aggregates of dermal basaloid tumor cells in the dermis. The outer layer of malignant cells is arranged in a longitudinal linear manner such that they are parallel to each other; this is referred to as peripheral palisading of the cells. In addition, a characteristic processing artifact can also often be observed in which there is retraction of the myxoid dermal stroma from the peripheral cell layer of the tumor nests resulting in the appearance of a clear cleft surrounding the neoplasm [1-3,8-10].

Several investigators have confirmed that basal cell carcinoma in situ is the second most common subtype of basal cell carcinoma; previously, this variant of the neoplasm has been referred to as superficial basal cell carcinoma [1-3,8-10]. Originally, the tumor was considered to be multifocal based on its pathologic appearance of apparently distinct tumor aggregates along the basal layer of the epidermis. However, subsequent studies have demonstrated that this subtype of basal cell carcinoma is unicentric and monoclonal based not only on three-dimensional reconstruction using serial sections and a computer which showed all the tumor foci to be interconnected, but also lack of loss of heterozygosity at the patched 1 (Ptch1) locus using polymerase chain reaction amplification of tumor deoxyribonucleic acid (DNA) [11,12].

The features of the patients in this report (summarized in Table 2) are typical of those observed in individuals with basal cell carcinoma in situ. The tumor frequently occurs on the trunk; however, it is not uncommonly found on either the flanks or back. Clinically, it appears as a superficial, thin, erythematous plaque, whose borders may be rolled, with or without overlying scale; the morphologic differential diagnosis includes dermatitis and squamous cell carcinoma in situ [1-3,8-10].

**Table 2:** Characteristics of patients with basal cell carcinoma in situ. ^a^Fitzpatrick skin type 1 refers to individuals with skin that always burns and never tans.

Features	Case 1	Case 2	Case 3
Age	43-year-old	63-year-old	69-year-old
Race	Caucasian	Caucasian	Caucasian
Skin type^a^	Fitzpatrick skin type 1	Fitzpatrick skin type 1	Fitzpatrick skin type 1
Gender	Male	Male	Male
Skin cancer history	None	Basal cell carcinoma, squamous cell carcinoma	None
Duration	Six months	Four months	Unknown
Location	Right upper abdomen	Lateral right lower abdomen	Left lower back
Symptoms	None	None	None
Morphology	Erythematous, triangular-shaped plaque	Erythematous, scaly plaque with peripheral nodules	Erythematous, focally crusted plaque with peripheral nodules
Size	5 x 4 x 4 cm	2 x 2 cm	4 x 2.5 cm
Treatment	Excision	5% imiquimod cream daily (five consecutive days each week for six weeks)	Excision
Follow-up	No recurrence: 15 months	No recurrence: 21 months	No recurrence: two years

Microscopic evaluation of the tissue biopsy specimen of a basal cell carcinoma in situ classically shows discrete areas of basaloid tumor cells predominantly restricted to the lower layers of the epidermis that are focally distributed along the basal epidermal layer; there is peripheral palisading of the outer layer of cancer cells which overlie the myxoid stroma of the papillary dermis and are separated from the dermis by artifactual cleft-like spaces. Occasionally, intraepidermal collections of tumor cells expand and nearly fill the epidermis similar to what is typically observed in squamous cell carcinoma in situ. Lymphocytic inflammation may also be present in the upper dermis beneath the in situ carcinoma [1-3,8-10].

Various treatment modalities have been used to successfully treat basal cell carcinoma in situ. In addition to surgical treatment using a scalpel (with either a routine excision or tumor removal using the Mohs micrographic technique), laser therapy (with either carbon dioxide laser or pulsed-dye laser), or curettage and desiccation, or cryosurgery with liquid nitrogen are all very effective therapeutic approaches for tumor management. However, the absence of tumor invasion also allows basal cell carcinoma in situ to be amendable to non-surgical and topical interventions such as photodynamic therapy following the application of photosensitizers and utilizing either 5% 5-fluorouracil cream or 5% imiquimod cream. In addition, albeit less frequently utilized, in situ basal cell carcinoma has also been found to be successfully treated with either intralesional interferon alpha or superficial radiotherapy [13-17].

Cutaneous basal cell carcinoma may have only one pathologic subtype. However, basal cell carcinoma of the skin may display mixed histology consisting of two or more pathologic variants; the tumor can contain only non-aggressive pathologic subtypes, only aggressive pathologic subtypes, or both. Hence, microscopic evaluation of a cutaneous basal cell carcinoma can demonstrate basal cell carcinoma in situ in the overlying epidermis and either nodular basal cell carcinoma (non-aggressive pattern) or infiltrating or morpheaform basal cell carcinoma (aggressive pattern) or both. Tumors with these features may provide additional insight regarding potential carcinoma recurrence when a superficial biopsy of the neoplasm only demonstrates basal cell carcinoma in situ [18].

Finally, basal cell carcinoma is not restricted to the skin. Indeed, basal cell carcinoma of the prostate is a rare prostate cancer subtype. Importantly, similar to the basal cell carcinoma in situ of the skin as the non-invasive variant of cutaneous basal cell carcinoma, basal cell carcinoma in situ of the prostate has recently been described [19,20].

## Conclusions

Basal cell carcinoma of the skin has several invasive subtypes in which either individual tumor cells and/or distinct nests of malignant basaloid cells that are not contiguous with the overlying epidermis appear in the underlying dermis. Similar to cutaneous squamous cell carcinoma in situ and cutaneous melanoma in situ, cutaneous basal cell carcinoma in situ demonstrates tumor cells that are restricted only to the epidermis without non-contiguous invasion of the neoplasm into the dermal stroma. Three men with typical features of basal cell carcinoma in situ of the skin are described in this case series; their skin cancer presented as a red plaque on the abdomen or back that was successfully treated without recurrence by surgical excision or topical 5% imiquimod cream. Indeed, similar to the patients in this report, morphologic features of an erythematous, scaly or non-scaly, and superficial plaque commonly on the trunk provide a characteristic correlation with cutaneous basal cell carcinoma in situ. Since the tumor is only localized to the overlying epidermis, several therapeutic modalities are available to successfully manage the cancer. However, if the neoplasm is a basal cell carcinoma of mixed histology with basal cell carcinoma in situ overlying a more aggressive pathologic subtype of basal cell carcinoma in the underlying dermis, a superficial skin biopsy may not discover the deeper carcinoma subtype and result in tumor persistence or recurrence if a more conservative approach to treatment is utilized. In summary, based on clinicopathologic correlation of morphology, histology, tumor biology, and response to treatment, neoplasms that were previously designated as superficial basal cell carcinoma are more appropriately classified as cutaneous basal cell carcinoma in situ.
